# *ERBB2*-Low Expression by Race and Ethnicity Among Patients With Triple-Negative Breast Cancer

**DOI:** 10.1001/jamanetworkopen.2025.14864

**Published:** 2025-06-11

**Authors:** Astrid Botty van den Bruele, Kerri-Anne Crowell, Samantha M. Thomas, Hannah E. Woriax, Kendra M. Parrish, Akiko Chiba, Laura H. Rosenberger, Ton Wang, Jennifer K. Plichta, Maggie L. DiNome

**Affiliations:** 1 Department of Surgery, Duke University School of Medicine, Durham, North Carolina; 2 Duke Cancer Institute, Duke University, Durham, North Carolina; 3 Department of Biostatistics and Bioinformatics, Duke University School of Medicine, Durham, North Carolina

## Abstract

**Importance:**

Racial and ethnic disparities in pathologic complete response (pCR) and overall survival (OS) have been reported in patients with triple-negative breast cancer (TNBC). In addition to socioeconomic factors and access to care, differences in tumor biology may affect outcomes. The association of *ERBB2-*low expression with outcomes in patients with TNBC has been minimally explored, including studies evaluating outcomes based on race and ethnicity.

**Objective:**

To investigate whether *ERBB2-*low expression among patients with TNBC varies by race and ethnicity and is associated with pCR and OS.

**Design, Setting, and Participants:**

This retrospective cohort study used the National Cancer Database, a national hospital-based tumor registry, from 2010 to 2019. Participants included adult female patients with stages I to III TNBC treated with neoadjuvant chemotherapy followed by surgery. Tumors with immunohistochemistry scores of 1+ or 2+, in situ hybridization nonamplified, were classified as *ERBB2-*low; those with immunohistochemistry scores of 0, as *ERBB2*-zero. Primary analysis was conducted from September 18 to October 18, 2023.

**Main Outcomes and Measures:**

Rates of *ERBB2*-low vs *ERBB2*-zero TNBC by race and ethnicity and pCR and OS by race and ethnicity and by *ERBB2* status.

**Results:**

Overall, 31 888 patients with TNBC were identified (median age, 53 [IQR, 44-61] years). Of those, 1078 patients (3.4%) were Asian; 7642 (24.0%), Black; 2578 (8.1%), Hispanic; 20 264 (63.5%), White; and 326 (1.0%), other. Over half of the study population (16 332 [51.2%]) displayed *ERBB2*-low expression and 15 556 (48.8%), *ERBB2*-zero expression. Patients with *ERBB2*-low expression demonstrated lower rates of pCR than those with *ERBB2*-zero expression (4675 [28.6%] vs 4787 [30.8%]; *P* < .001); *ERBB2*-low expression was associated with lower odds of pCR after adjustment for patient, socioeconomic, and tumor variables (odds ratio, 0.93; 95% CI, 0.88-0.99; *P* = .01). When patients were stratified by race and ethnicity, Hispanic patients had higher rates of *ERBB2*-zero expression than *ERBB2*-low expression (1391 of 2578 [54.0%] vs 1187 of 2578 [46.0%]; *P* < .001); no differences were noted among other racial and ethnic groups. Hispanic patients also had higher rates of pCR among those with *ERBB2*-low (Hispanic, 30.7%; Asian, 24.6%; Black, 29.5%; White, 27.1%) and *ERBB2*-zero (Hispanic, 33.2%; Asian, 32.1%; Black, 27.1%; White, 31.7%) TNBC. Non-Hispanic Asian patients had the highest 60-month OS among both those with *ERBB2*-zero (79%; 95% CI, 75%-83%) and *ERBB2*-low expression (84%; 95% CI, 80%-87%). Among Asian and White patients, *ERBB2*-low expression was associated with prolonged OS compared with *ERBB2*-zero expression (hazard ratios, 0.69 [95% CI, 0.50-0.95] [*P* = .02] and 0.85 [95% CI, 0.79-0.92] [*P* < .001], respectively). Black patients had lower pCR and OS rates regardless of *ERBB2* status.

**Conclusions and Relevance:**

In this cohort study of patients with TNBC, *ERBB2*-low expression and its association with clinical outcomes varied across racial and ethnic groups. These findings highlight the need for further research to understand the biological implications of *ERBB2*-low expression in diverse populations of patients with TNBC.

## Key Points

**Question:**

Does *ERBB2*-low expression and its association with clinical outcomes vary by race and ethnicity among patients with triple-negative breast cancer (TNBC)?

**Findings:**

In this cohort study of 31 888 patients with TNBC from the National Cancer Database, Hispanic patients had higher rates of *ERBB2*-zero expression (54.0%) than *ERBB2*-low expression (46.0%), a significant difference, and higher rates of pathologic complete response to neoadjuvant chemotherapy. In addition, *ERBB2*-low expression was associated with lower rates of pathologic complete response.

**Meaning:**

These findings suggest that *ERBB2*-low expression and its association with clinical outcomes vary across racial and ethnic groups in patients with TNBC, a potentially relevant finding that should be considered in future clinical studies.

## Teaser

This cohort study assesses whether *ERBB2*-low status and its association with pathologic complete response and overall survival differ by race and ethnicity among patients with triple-negative breast cancer.

## Introduction

*ERBB2*-negative breast cancer (BC) lacks overexpression of ErbB2 or amplification of the *ERBB2* gene defined by an immunohistochemistry (IHC) score of 0 to 1+ or 2+, with negative in situ hybridization (ISH) findings.^1,2^ This distinction defines an invasive BC that does not respond clinically to traditional anti-*ERBB2* therapies based on the NSABP (National Surgical Adjuvant Breast and Bowel Project) B-47 study.^3^ However, recent novel *ERBB2*-directed therapies have demonstrated efficacy in BC with low expression of *ERBB2*.^4^ The DESTINY-Breast04 randomized clinical trial demonstrated that antibody-drug conjugate (ADC) therapy significantly improved progression-free and overall survival (OS) for patients with *ERBB2*-low metastatic BC. The trial reported a 50% decrease in disease progression and a 36% reduction in mortality, independent of hormone receptor (HR) status.^4^ Based on these findings, low expression of *ERBB2* defined by an IHC score of 1+ or 2+, ISH nonamplified, has become a predictive biomarker for response to currently available ADC therapies in the metastatic setting.

Data are mixed, however, regarding the prognostic relevance of *ERBB2*-low BC as a unique BC subtype. Some studies^5,6,7,8,9^ have suggested that HR status is the primary driver of tumor biology rather than *ERBB2* status, with no differences noted in OS based on *ERBB2* status. Additionally, *ERBB2*-low expression appears to be associated with HR status, with patients with HR-positive invasive BC demonstrating a higher frequency of *ERBB2-*low tumors than those with HR-negative BC.^5,7,9^ A recent meta-analysis,^10^ however, demonstrated an improved OS for patients with *ERBB2-*low BC in both the early and metastatic settings. In addition, a pooled analysis of 4 neoadjuvant studies^11^ demonstrated that patients with *ERBB2-*low BC had improved OS compared with patients with *ERBB2-*zero BC, and this was noted in a subgroup with triple-negative breast cancer (TNBC) as well. Studies focusing on TNBC have also suggested improved BC-specific survival in patients with *ERBB2*-low BC^12^; moreover, among patients with TNBC who did not achieve a pCR, *ERBB2*-low expression was associated with an improved prognosis.^11,12^

Several studies have demonstrated that outcomes for patients with TNBC vary by race and ethnicity,^13,14,15,16,17,18,19^ but the significance of *ERBB2*-low expression based on race and ethnicity in patients with TNBC has yet to be examined. Although inclusive of both HR-positive and HR-negative subtypes, the DESTINY-Breast04 study included a small minority of Black (1.8%) and Hispanic (3.8%) patients,^4^ so the significance of *ERBB2*-low expression and efficacy of ADC therapy for patients in these underrepresented groups is unclear. Members of our group and other investigators^14,18,19^ have demonstrated that race and ethnicity is a significant variable associated with treatment response in patients with TNBC. Studies have also shown differing rates of response by *ERBB2* status, with lower rates of pCR consistently seen with *ERBB2*-low BC, although rates by HR status have been conflicting.^5,6,7,8,10,11,20,21,22^ The extent to which low *ERBB2* expression contributes to the observed disparities in clinical outcomes among patients with TNBC by race and ethnicity is currently unknown.

In this study, we examined the hypothesis that *ERBB2* status and the association with pCR and OS differ among patients with TNBC based on race and ethnicity. To address this, we evaluated a large cohort of patients with TNBC from the National Cancer Database (NCDB) who received neoadjuvant chemotherapy (NAC) followed by surgery and assessed clinicopathologic variables and outcomes by race and ethnicity and *ERBB2* status.

## Methods

The data for this retrospective cohort study were sourced from the NCDB, a national hospital-based tumor registry including approximately 80% of BC diagnosed annually.^23^ The present study was granted exemption and waiver of informed consent by the Duke University Institutional Review Board per the Code of Federal Regulations (45 CFR §46.104). This study adhered to the Strengthening the Reporting of Observational Studies in Epidemiology (STROBE) guidelines for reporting observational research.

### Study Design and Patient Population

Adult female patients (aged ≥18 years) diagnosed from 2010 to 2019, with clinical stages I to III TNBC treated with NAC followed by surgery were identified from the 2004-2019 NCDB (2019 Participant User File). The study population was restricted to individuals diagnosed from 2010 and after because 2010 was the year *ERBB2* status became a required data field. Patients without more granular *ERBB2* data (IHC and/or ISH results), race and ethnicity data, or surgical pathology data or with unknown or missing data were excluded. Additional exclusion criteria included clinical stage 0 or IV disease, bilateral BC, male BC, or no NAC (eFigure 1 in Supplement 1). Estrogen receptor, progesterone receptor, and *ERBB2* negativity were defined according to American Society of Clinical Oncology and College of American Pathologists guidelines.^1,24^ *ERRB2* status was further categorized into *ERBB2*-zero (IHC 0) or *ERBB2*-low (IHC 1+ or 2+, ISH nonamplified). pCR was defined as no residual invasive disease in the breast and lymph nodes (ypTis or ypT0 and ypN0). All other findings were defined as residual disease (RD).

Race and ethnicity were categorized as Hispanic, non-Hispanic Asian (Asian), non-Hispanic Black (Black), non-Hispanic White (White), and non-Hispanic other (other), which includes American Indian or Alaska Native, Native Hawaiian or Other Pacific Islander, and other (eMethods in the Supplement). Participating cancer centers entered race and ethnicity data according to STORE (Standards for Oncology Registry Entry) guidelines based on self-reported patient data.

### Statistical Analysis

Primary analysis was conducted from September 18 to October 18, 2023. Patient, disease, and treatment characteristics were summarized by number (percentage) for categorical and median (IQR) for continuous variables by *ERBB2* status (*ERBB2*-zero or *ERBB2*-low). Differences across groups were tested using the χ^2^ test for categorical variables and independent 2-sample *t* tests or Mann-Whitney tests for continuous variables. The Kaplan-Meier method was used to estimate unadjusted OS and log-rank tests for differences in survival between groups. OS was defined as the time from diagnosis to death due to any cause. Surviving patients were censored at date of last follow-up (ending in 2021). The reverse Kaplan-Meier method was used to estimate follow-up time. A Cox proportional hazards model was used to estimate the association of *ERBB2* status with OS after adjustment for available covariates, including demographic, socioeconomic, and tumor variables. This model included a sandwich covariance estimator to account for the correlation of patients treated at the same facility. The proportional hazards assumption was evaluated using both graphical and statistical approaches. For categorical covariates, Kaplan-Meier survival curves and log-log survival plots were used to assess the proportional hazards assumption. For continuous covariates, the supremum test was applied, and time-varying effects were assessed by including an interaction term between the covariate and the log-transformed survival time (log[time]) in the Cox proportional hazards regression model. The significance of the interaction term was evaluated using a Wald χ^2^ test. The proportional hazards assumption held for all covariates except for tumor size. To account for this violation, we included an interaction between tumor size and log(time). The proportional hazards assumptions were tested for all subgroup models separately. Similarly, tumor size violated the proportional hazards assumption for the subgroup analyses of White and Black patients, and an interaction term was included in these models.

Due to NCDB administrative censoring, patients diagnosed in 2019 were excluded from survival analyses. A logistic regression model was used to estimate the association of select covariates with achievement of pCR in each cohort. Additional interaction analyses were also conducted. Only patients with complete data were included in each analysis; effective sample sizes are included for each Table and Figure. No adjustments were made for multiple comparisons. All statistical analyses were conducted using SAS, version 9.4 (SAS Institute Inc) and R Studio, version 4.2.2 (R Program for Statistical Computing). Two-sided *P* < .05 indicated statistical significance.

## Results

### Patient and Tumor Characteristics

In total, 31 888 female patients with TNBC (median patient age, 53 [IQR, 44-61] years) met study criteria. Of those, 1078 patients (3.4%) were Asian (median age, 51 [IQR, 41-61] years); 7642 (24.0%), Black (median age, 52 [IQR, 44-60] years); 2578 (8.1%), Hispanic (median age, 47 [IQR, 39-55] years); 20 264 (63.5%), White (median age, 54 [IQR, 45-63] years); and 326 (1.0%), other race (median age, 47 [IQR, 39-57] years). In total, 15 556 patients (48.8%) were categorized as *ERBB2*-zero and 16 332 (51.2%) as *ERBB2*-low (Table 1). Most patients had invasive ductal carcinoma (28 534 [89.5%]) and poorly differentiated histologic findings (25 855 of 30 315 [85.3%]). More than half were 50 years or older (19 019 [59.6%]) and had clinical T2 disease (17 105 [53.6%]) and/or clinical N0 disease (16 397 [51.4%]). Younger patients (aged <50 years) had slightly higher rates of *ERBB2*-zero expression than *ERBB2*-low expression (6527 of 12 869 [50.7%] vs 6342 of 12 869 [49.3%]). Clinical T category did not differ between groups, but those with *ERBB2*-low expression had higher rates of lymph node positivity (8148 [49.9%] vs 7343 [47.2%]; *P* < .001) and fewer high-grade tumors (13 083 [84.2%] vs 12 772 [86.4%]; *P* < .001) (Table 1). Median follow-up time for all patients was 53.7 (95% CI, 53.3-54.2) months.

**Table 1.** Patient Demographic and Tumor Characteristics Among Patients With Triple-Negative Breast Cancer in the National Cancer Database

Characteristic	Patient group by *ERBB2* status, No. (%)^a^			*P* value
	All (N = 31 888)	*ERBB2*-zero (n = 15 556)	*ERBB2*-low (n = 16 332)	
Age at diagnosis, median (IQR), y	53.0 (44.0-61.0)	52.0 (43.0-61.0)	53.0 (44.0-62.0)	<.001
Age group, y				
<50	12 869 (40.4)	6527 (42.0)	6342 (38.8)	<.001
≥50	19 019 (59.6)	9029 (58.0)	9990 (61.2)	
Charlson Deyo Comorbidity Score				
0	27 407 (85.9)	13 400 (86.1)	14 007 (85.8)	.25
1	3545 (11.1)	1687 (10.8)	1858 (11.4)	
≥2	936 (2.9)	469 (3.0)	467 (2.9)	
Race and ethnicity				
Asian	1078 (3.4)	529 (3.4)	549 (3.4)	<.001
Black	7642 (24.0)	3718 (23.9)	3924 (24.0)	
Hispanic	2578 (8.1)	1391 (8.9)	1187 (7.3)	
White	20 264 (63.5)	9769 (62.8)	10 495 (64.3)	
Other^b^	326 (1.0)	149 (1.0)	177 (1.1)	
Clinical T category				
cT0 or in situ	102 (0.3)	52 (0.3)	50 (0.3)	.15
cT1	6595 (20.7)	3303 (21.2)	3292 (20.2)	
cT2	17 105 (53.6)	8320 (53.5)	8785 (53.8)	
cT3	5092 (16.0)	2446 (15.7)	2646 (16.2)	
cT4	2994 (9.4)	1435 (9.2)	1559 (9.5)	
Clinical N category				
cN0	16 397 (51.4)	8213 (52.8)	8184 (50.1)	<.001
cN1	11 608 (36.4)	5488 (35.3)	6120 (37.5)	
cN2	2172 (6.8)	1060 (6.8)	1112 (6.8)	
cN3	1711 (5.4)	795 (5.1)	916 (5.6)	
Histology				
Ductal	28 534 (89.5)	13 811 (88.8)	14 723 (90.1)	<.001
Lobular	1238 (3.9)	604 (3.9)	634 (3.9)	
Other	2116 (6.6)	1141 (7.3)	975 (6.0)	
Grade^c^				
1	236 (0.8)	105 (0.7)	131 (0.8)	<.001
2	4224 (13.9)	1904 (12.9)	2320 (14.9)	
3	25 855 (85.3)	12 772 (86.4)	13 083 (84.2)	
Overall response				
pCR	9462 (29.7)	4787 (30.8)	4675 (28.6)	<.001
RD	22 426 (70.3)	10 769 (69.2)	11 657 (71.4)	

Abbreviations: pCR, pathologic complete response; RD, residual disease.

^a^ Data were from 2010 to 2019. Percentages may not total 100 due to rounding.

^b^ Includes American Indian or Alaska Native, Native Hawaiian or Other Pacific Islander, and other.

^c^ Data were missing for 1573 patients.

### *ERBB2* Status by Race and Ethnicity and Association With pCR

**Figure 1.**
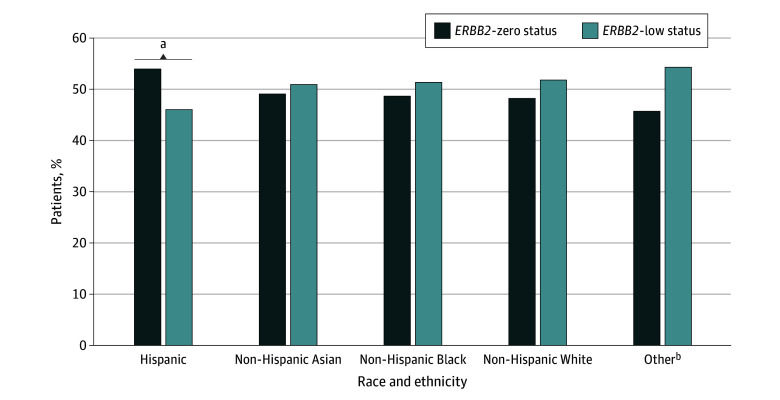
*ERBB2* Status Stratified by Race and Ethnicity Among Patients With Triple-Negative Breast Cancer in the National Cancer Database (2010-2019) ^a^*P* < .001. ^b^Includes American Indian or Alaska Native, Native Hawaiian or Other Pacific Islander, and other.

When evaluating differences in *ERBB2* status by race and ethnicity, Hispanic patients (n = 2578) had significantly higher rates of *ERBB2*-zero than *ERBB2*-low expression (1391 of 2578 [54.0%] vs 1187 of 2578 [46.0%]; *P* < .001); all other races and ethnicities had higher rates of *ERBB2*-low expression, but these differences were not statistically significant (Figure 1). The association of Hispanic race and ethnicity with *ERBB2*-low TNBC remained after adjustment for demographic, socioeconomic, and tumor variables (odds ratio [OR], 0.84; 95% CI, 0.76-0.92; *P* = .003) (eTable 1 in Supplement 1). Rates of pCR differed by *ERBB2* status, with patients with *ERBB2*-zero TNBC demonstrating higher rates of overall pCR (4787 [30.8%] vs 4675 [28.6%]; *P* < .001) (Table 1), and nodal pCR (2870 of 7343 [39.1%] vs 2906 of 8148 [35.7%]; *P* < .001) compared with patients with *ERBB2*-low TNBC. After adjustment for covariates, *ERBB2*-low status remained associated with lower rates of pCR (OR, 0.93; 95% CI, 0.88-0.99; *P* = .01) (Table 2). Among those with *ERBB2*-low expression, Hispanic patients had higher rates of pCR (365 of 1187 [30.7%]) compared with other racial and ethnic groups (135 of 549 [24.6%] for Asian patients; 1032 of 3924 [26.3%] for Black patients; 3095 of 10 495 [29.5%] for White patients; 48 of 177 [27.1%] for patients of other race) (eTable 2 in Supplement 1). Both Hispanic patients (*ERBB2*-zero expression, 462 of 1391 [33.2%]; *ERBB2*-low expression, 365 of 1187 [30.7%]; *P* = .001) and Asian patients (*ERBB2*-zero expression, 170 of 529 [32.1%]; *ERBB2*-low expression, 135 of 549 [24.6%]; *P* = .045) had significantly different pCR rates based on *ERBB2* status; no differences were seen in patients of Black, White, or other race and ethnicity (eTable 2 in Supplement 1). After adjustment, Hispanic patients had higher rates of pCR (OR, 1.18; 95% CI, 1.06-1.32; *P* = .003), while Black patients had lower rates (OR, 0.88; 95% CI, 0.82-0.95; *P* = .001) (Table 2) compared with White patients. The association of race and ethnicity with odds of achieving pCR did not differ by *ERBB2* status (interaction *P* = .17).

**Table 2.** Adjusted Logistic Regression for Overall Response Among Patients With Triple-Negative Breast Cancer in the National Cancer Database^a^

Variable	OR (95% CI)	*P* value	Overall *P* value
ERBB2 status			
Zero	1 [Reference]	NA	.01
Low expression	0.93 (0.88-0.99)	.01	
Race and ethnicity			
White	1 [Reference]	NA	<.001
Asian	0.90 (0.77-1.06)	.21	
Black	0.88 (0.82-0.95)	.001	
Hispanic	1.18 (1.06-1.32)	.003	
Other^b^	0.95 (0.71-1.27)	.73	
Age, y			
≥50	1 [Reference]	NA	<.001
<50	1.28 (1.20-1.35)	<.001	
Charlson Deyo Comorbidity Score			
0	1 [Reference]	NA	.01
1	0.91 (0.83-1.00)	.05	
≥2	0.77 (0.63-0.93)	.01	
Clinical T category			
cT1	1 [Reference]	NA	<.001
cT0/is	0.56 (0.21-1.50)	.25	
cT2	1.03 (0.95-1.11)	.47	
cT3	0.74 (0.66-0.83)	<.001	
cT4	0.60 (0.52-0.70)	<.001	
Clinical N category			
cN0	1 [Reference]	NA	<.001
cN1	0.89 (0.84-0.95)	<.001	
cN2	0.73 (0.64-0.83)	<.001	
cN3	0.86 (0.75-0.99)	.03	
Grade			
1	1 [Reference]	NA	<.001
2	1.40 (0.92-2.14)	.11	
3	2.55 (1.69-3.86)	<.001	

Abbreviations: NA, not applicable; OR, odds ratio.

^a^ Data were from 2010 to 2019 for 24 424 patients and 7046 events. Analyses were adjusted for income, educational level, insurance status, community type, facility type and location, surgery type, tumor size, and time to treatment from chemotherapy to surgery.

^b^ Includes American Indian or Alaska Native, Native Hawaiian or Other Pacific Islander, and other.

### *ERBB2* Status by Race and Ethnicity and Association With OS

**Figure 2.**
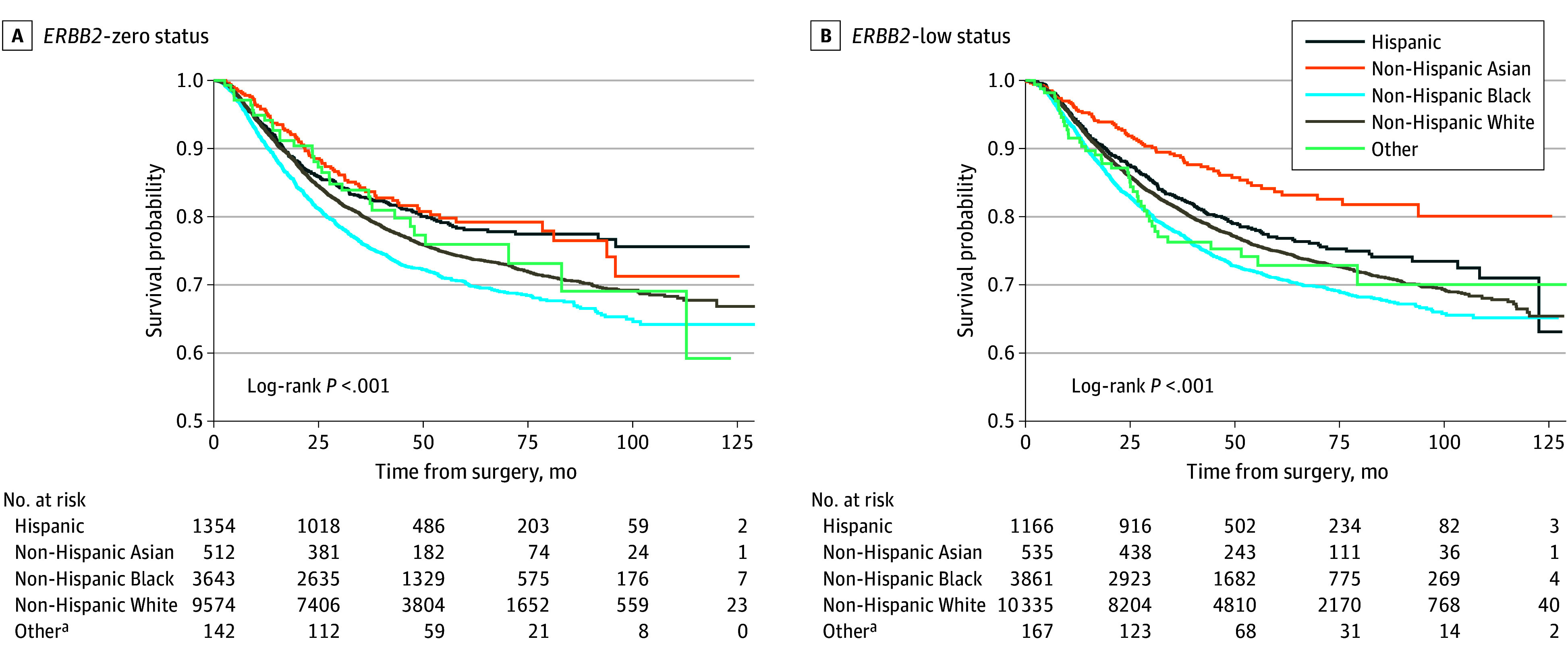
Unadjusted Overall Survival by Race and Ethnicity Stratified for *ERRB2* Status Among Patients With Triple-Negative Breast Cancer in the National Cancer Database (2010-2019) ^a^Includes American Indian or Alaska Native, Native Hawaiian or Other Pacific Islander, and other.

Unadjusted OS did not differ by *ERBB2* status (eFigure 2 in Supplement 1). However, among patients with *ERBB2*-low expression, when stratified by race and ethnicity, Asian patients had higher proportions of OS (84%; 95% CI, 80%-87%) at 60 months; among those with *ERBB2*-zero expression, Asian patients similarly had higher OS (79%; 95% CI, 75%-83%) at 60 months. However, Hispanic patients demonstrated more favorable long-term survival among those with *ERBB2*-zero expression, with survival probabilities exceeding those of non-Hispanic Asian patients during extended follow-up (Figure 2). This is further supported by 120-month survival estimates in the *ERBB2*-zero group, where Hispanic patients had higher overall survival (76%; 95% CI, 72%-79%) compared with non-Hispanic Asian patients (71%; 95% CI, 62%-79%) (eTable 3 in Supplement 1). Black patients had the lowest OS in both groups at 60 months (*ERBB2*-zero expression, 71% [95% CI, 69%-72%]; *ERBB2*-low expression, 71% [95% CI, 69%-73%]) (Figure 2 and eTable 3 in Supplement 1). Additionally, among Asian patients, those with *ERBB2*-low expression had higher unadjusted OS than those with *ERBB2*-zero expression (eg, at 60 months, 84% [95% CI, 80%-87%] vs 79% [95% CI, 75%-83%]) (eFigure 3 in Supplement 1). The association of race and ethnicity with OS differed for those achieving pCR vs those with RD by *ERBB2* status (interaction *P* = .03). Among those with *ERBB2*-low expression and pCR, no difference in OS was noted by race and ethnicity; among those with RD, Asian patients had higher OS. Among those with *ERBB2*-zero expression, Hispanic patients had the highest OS regardless of treatment response (eFigure 4 in Supplement 1).

After adjustment for covariates, including receipt of adjuvant radiotherapy, both race and ethnicity and *ERBB2* status remained associated with OS. Hispanic patients (hazard ratio, 0.72; 95% CI, 0.63-0.81; *P* < .001) and Asian patients (hazard ratio, 0.65; 95% CI, 0.54-0.78; *P* < .001) had improved OS compared with White patients; *ERBB2*-low status was also associated with improved OS (hazard ratio, 0.88; 95% CI, 0.83-0.93; *P* < .001) (Table 3). The association of race and ethnicity with OS did not differ by treatment response (interaction *P* = .05) or by *ERBB2* status (interaction *P* = .49). Interestingly, a subgroup analysis by race and ethnicity demonstrated that the ability to achieve a pCR among Hispanic patients (n = 1899) was associated with even larger improvement in OS (hazard ratio, 0.17; 95% CI, 0.11-0.26; *P* < .001) (eTable 4 in Supplement 1) compared with that seen in the study cohort overall (hazard ratio, 0.25; 95% CI, 0.23-0.28; *P* < .001). After adjustment for patient, socioeconomic, and tumor variables, *ERBB2* status was not associated with OS among Hispanic patients. However, the association of achieving pCR with OS was different based on *ERBB2* status (interaction *P* = .046). *ERBB2*-low expression was associated with improved OS among Asian patients (hazard ratio, 0.69; 95% CI, 0.50-0.95; *P* = .02) and White patients (hazard ratio, 0.85; 95% CI, 0.79-0.92; *P* < .001) (eTable 4 in Supplement 1).

**Table 3.** Adjusted Overall Survival Among Patients With Triple-Negative Breast Cancer in the National Cancer Database^a^

Variable	HR (95% CI)	*P* value	Overall *P* value
*ERBB2* status			
Zero	1 [Reference]	NA	<.001
Low	0.88 (0.83-0.93)	<.001	
Overall response			
RD	1 [Reference]	NA	<.001
pCR	0.29 (0.26-0.32)	<.001	
Race and ethnicity			
Non-Hispanic White	1 [Reference]	NA	<.001
Hispanic	0.72 (0.63-0.81)	<.001	
Non-Hispanic Asian	0.65 (0.54-0.78)	<.001	
Non-Hispanic Black	1.02 (0.96-1.10)	.50	
Other^b^	0.97 (0.71-1.32)	.82	
Age, y			
≥50	1 [Reference]	NA	.002
<50	0.91 (0.85-0.96)	.002	
Charlson Deyo Comorbidity Score			
0	1 [Reference]	NA	<.001
1	1.17 (1.08-1.27)	<.001	
≥2	1.40 (1.22-1.61)	<.001	
Tumor size, cm	1.48 (1.47-1.50)	<.001	<.001
Clinical T category			
cT1	1 [Reference]	NA	<.001
cT0/is	1.39 (0.78-2.46)	.16	
cT2	2.06 (1.89-2.25)	<.001	
cT3	5.29 (4.77-5.86)	<.001	
cT4	5.15 (4.62-5.73)	<.001	
Clinical N category			
cN0	1 [Reference]	NA	<.001
cN1	1.73 (1.62-1.85)	<.001	
cN2	2.17 (1.96-2.39)	<.001	
cN3	2.38 (2.14-2.65)	<.001	
Grade			
1	1 [Reference]	NA	.004
2	1.24 (0.87-1.76)	.24	
3	1.39 (0.98-1.97)	.07	
Treatment with radiotherapy			
No	1 [Reference]	NA	.01
Yes	1.09 (1.02-1.17)	.01	
Surgery type			
Mastectomy	1 [Reference]	NA	<.001
Lumpectomy	0.65 (0.61-0.70)	<.001	
Weeks from diagnosis to surgery	1.00 (0.99-1.00)	.05	.05

Abbreviations: HR, hazard ratio; NA, not applicable; pCR, pathologic complete response; RD, residual disease.

^a^ Data were from 2010 to 2019 for 22 741 patients and 5281 events. Model includes a time-dependent interaction between tumor size and log(time); HR for interaction was 0.82 (95% CI, 0.82-0.83; *P* < .001). Additional covariates include educational level, income level, insurance type, facility type, facility location, and community type.

^b^ Includes American Indian or Alaska Native, Native Hawaiian or Other Pacific Islander, and other.

## Discussion

The heterogeneity of TNBC, defined solely by the lack of 3 traditional targets, makes it arguably the least understood BC subtype and most difficult to treat. Although survival rates for patients with BC have improved overall, patients with TNBC continue to have higher rates of distant relapse and worse OS than any other BC subtype.^25^ Additionally, patients with TNBC exhibit disparities in outcomes based on race and ethnicity, with differences noted in both OS and rates of response to standard chemotherapy.^14,15,17,18,19,26^ Recently, ADC therapy has been approved for treatment of patients with *ERBB2*-low BC, including those with TNBC, in the metastatic setting based on improved survival outcomes^4^; however, few patients of Black (1.8%) and Hispanic (3.8%) race and ethnicity were included in that study population. Therefore, variability in outcomes based on race and ethnicity for patients with *ERBB2*-low BC could not be assessed. In this retrospective cohort study, we focused our analysis on the association of *ERBB2*-low expression with clinical outcomes in patients with early-stage TNBC based on race and ethnicity.

Our findings suggest that *ERBB2* status differs by race and ethnicity in patients with TNBC, and this may contribute to the disparities in outcomes noted. Of considerable interest, we found that Hispanic patients had higher rates of *ERBB2*-zero TNBC than *ERBB2*-low TNBC, which is unlike the higher rates of *ERBB2*-low BC seen overall in our study and across other studies.^4,5,6,7^ Although few studies have assessed the prevalence of *ERBB2*-low BC by race and ethnicity, Peiffer et al^5^ similarly demonstrated that Hispanic patients had lower odds of *ERBB2*-low BC (OR, 0.85; 95% CI, 0.83-0.86; *P* < .001) compared with White patients. This distinction is notable, as data support that *ERBB2*-low tumors demonstrate a poorer response to NAC than *ERBB2*-zero tumors.^5,7,10,11,20,27^ The higher rates of pCR noted in Hispanic patients may reflect the higher percentage of *ERBB2*-zero TNBC seen in Hispanic patients in our study. The racial and ethnic disparity in *ERBB2* status is a novel finding that may provide insight into the differential response to NAC seen in Hispanic patients with TNBC.

Interestingly, recent data suggest that among patients with TNBC, *ERBB2*-zero expression may have a more immune-active phenotype compared with *ERBB2*-low expression,^28,29,30^ and this may influence the response of this biological subtype to treatment. We and other previous investigators^28,30^ have shown a lower density of tumor-infiltrating lymphocytes with *ERBB2*-low TNBC; this association is important given that several studies^31,32,33^ have demonstrated that a higher density of tumor-infiltrating lymphocytes correlates with pCR in patients with TNBC treated with NAC. Ortiz Valdez et al^34^ demonstrated that among Hispanic patients from Mexico with TNBC, the most frequently identified subtype was immunomodulatory, and this immune-enriched subtype was associated with the highest rates of pCR in their study. A recent study by Ng’ang’a et al^35^ assessed response to chemotherapy-immunotherapy per the KEYNOTE 522 regimen and also demonstrated higher rates of pCR among Hispanic patients. Studies have shown that immune-active tumors exhibit better objective response rates to immunotherapy than immune-depleted tumors.^36,37^ Although their sample size was small (n = 29), their results suggest disparities in response to chemotherapy-immunotherapy by race and ethnicity as well. Our preliminary findings support that TNBC among Hispanic patients, among whom the majority of tumors have *ERBB2*-zero expression, may be more immune active, resulting in higher rates of pCR. The novel finding in our study of a higher proportion of *ERBB2*-zero BC in Hispanic patients with TNBC needs to be further explored.

The existing data regarding the significance of *ERBB2*-low BC as a unique prognostic subtype are mixed.^5,6,7,10,11^ However, when assessed specifically by race and ethnicity, we see that *ERBB2* status may have prognostic relevance, at least in patients with TNBC. Our data show that Asian patients with *ERBB2*-low TNBC had prolonged OS compared with those with *ERBB2*-zero disease. Although no difference in OS was noted by race and ethnicity among patients with TNBC who achieved pCR,^19^ we did observe a difference in OS in our study among those who achieved pCR when assessed by *ERBB2* status: Hispanic patients had improved OS among those with *ERBB2*-zero expression. Furthermore, Hispanic patients demonstrated improved OS even among those with RD. For those with *ERBB2*-low TNBC, no survival difference was noted among those who achieved pCR based on race and ethnicity. However, among those with RD, Asian patients had markedly improved OS compared with other racial and ethnic groups. While the prognostic significance of *ERBB2*-low BC remains unclear, our study suggests that *ERBB2* status may be associated with survival outcomes in TNBC, particularly for Asian and Hispanic patients.

Interestingly, Asian patients with *ERBB2*-low BC had improved OS compared with those with *ERBB2*-zero BC, despite Asian patients with *ERBB2*-low BC having the lowest rates of pCR. This observation of lower pCR but improved OS mirrors that of luminal invasive BC in which RD after NAC does not impart a poorer prognostic outcome.^38,39^ In general, higher rates of pCR are seen with basal-like tumors, which account for the majority of TNBC; however, among TNBC, PAM50 subtyping has shown that increasing levels of *ERBB2* expression correlate with lower rates of basal-like tumors (*ERBB2* IHC 0, −85.2%; *ERBB2* IHC 1+, −85.4%; *ERBB2* IHC 2+, −78.4%).^6^ In a population of patients from China with TNBC, Dai et al^40^ demonstrated that more than 30% of patients with *ERBB2*-low TNBC had a non–basal-like phenotype compared with those with *ERBB2*-zero TNBC (30.3% vs 3.7%; *P* = .005). In addition, 61.5% of the non–basal-like *ERBB2*-low BC harbored a *PIK3Ca* variation,^40^ which is a commonly identified variation in as many as 40% of luminal BC. Jiang et al^41^ similarly demonstrated higher rates of *PIK3Ca* alterations in Asian patients with TNBC. These data and the results of our study suggest that *ERBB2*-low BC among Asian patients may be a unique prognostic subtype with more luminal features conferring a more favorable survival outcome for patients with TNBC. However, the impact of variables such as body mass index, which may contribute to differences in survival outcomes based on race and ethnicity,^42,43^ could not be evaluated in our study.

While our results suggest the relevance of *ERBB2* status among Hispanic and Asian patients, we did not observe an association between *ERBB2* status and outcomes in Black patients with TNBC. In fact, after adjustment for socioeconomic and clinicopathologic variables, including *ERBB2* status, Black race was still associated with lower odds of pCR. Additionally, in both *ERBB2* groups, Black patients had the lowest OS. Given that fewer than 2% of patients in the DESTINY-Breast04 study were of Black,^4^ it is unclear whether *ERBB2*-low expression is a reliable biomarker for response to ADC therapy in Black patients. A subgroup analysis of Asian patients, however, who represented nearly 40% of the study participants, did demonstrate an improvement in survival with ADC therapy among Asian patients with *ERBB2*-low metastatic BC.^44^ Certainly, the interaction among race and ethnicity, tumor biology, and outcomes is complex and warrants further study. Additionally, with DESTINY-Breast06 demonstrating response to ADC therapy of *ERBB2*-ultralow HR-positive BC (defined as IHC 0 with membrane staining),^45^ the significance of *ERBB2*-ultralow expression in patients with TNBC warrants future investigation.

### Limitations

This study has limitations. Relying on self-reported racial and ethnicity data from the NCDB limits our ability to account for genetic ancestry and mixed-race identities. Additionally, relevant variables like menopausal status, genetic mutations, body mass index, and many elements of social and structural determinants of health are not reported in the database. While the NCDB is the largest national tumor registry in the US, it is not population based; rather, hospitals participating in the NCDB are Commission on Cancer–accredited facilities, and therefore, results may not be generalizable. Furthermore, the NCDB does not capture systemic therapy regimens or treatment doses, which can influence survival. Finally, given the nature of the NCDB, there is no central pathology review, and the heterogeneity of *ERBB2* expression within a tumor and interobserver variability may have led to misclassification of *ERBB2* status.^2,46^

## Conclusions

In this cohort study of patients with TNBC, *ERBB2*-low expression and its association with clinical outcomes varied across racial and ethnic groups. Our research underscores disparities in outcomes based on *ERBB2* expression among Hispanic and Asian patients with TNBC, offering fresh perspectives on the significance of low *ERBB2* expression in these understudied populations. Our findings reiterate the critical need to recruit for racial and ethnic diversity in clinical trials and to focus future research on understanding the differences in tumor biology based on race and ethnicity that affect response to treatment and survival. This knowledge will facilitate tailored therapeutic strategies to improve outcomes for all patients with TNBC.

## Notes

Supplement 1. **eFigure 1.** Flowchart of Cohort of Patients With TNBC **eMethods.** Race and Ethnicity Reporting in NCDB **eTable 1.** Logistic Regression Predicting Low vs Zero *ERBB2* Expression (25 843 Patients;13 130 Events) Among Patients With TNBC in the NCDB (2010-2019) **eTable 2.** Rates of Pathologic Complete Response by *ERBB2* Status Within Race and Ethnicity **eFigure 2.** Unadjusted Overall Survival by *ERBB2* Status Among Patients With TNBC in the NCDB (2010-2019) **eTable 3.** Total Number of Patients for Each Race and Ethnicity by *ERBB2* Status and Number of Deaths and Censored Percent With Survival Rate **eFigure 3.** Unadjusted Overall Survival by *ERBB2* Status Within Race and Ethnicity Among Patients With TNBC in the NCDB (2010-2019) **eFigure 4.** Unadjusted Overall Survival by Race and Ethnicity Stratified for *ERBB2* Status and Overall Response **eTable 4.** Adjusted Overall Survival Among All Patients With TNBC Stratified by Race and Ethnicity

Supplement 2.
Data Sharing Statement
