# Supplementary material for: ERBB2-Low Expression by Race and Ethnicity Among Patients With Triple-Negative Breast Cancer
Source: JAMA Netw Open. 2025 Jun 11;8(6):e2514864. doi: 10.1001/jamanetworkopen.2025.14864 (PMC12159773; doi:10.1001/jamanetworkopen.2025.14864)
Supplement: Supplement 1. — eFigure 1. Flowchart of Cohort of Patients With TNBC eMethods. Race and Ethnicity Reporting in NCDB eTable 1. Logistic Regression Predicting Low vs Zero ERBB2 Expression (25 843 Patients;13 130 Events) Among Patients With TNBC in the NCDB (2010-2019) eTable 2. Rates of Pathologic Complete Response by ERBB2 Status Within Race and Ethnicity eFigure 2. Unadjusted Overall Survival by ERBB2 Status Among Patients With TNBC in the NCDB (2010-2019) eTable 3. Total Number of Patients for Each Race and Ethnicity by ERBB2 Status and Number of Deaths and Censored Percent With Survival Rate eFigure 3. Unadjusted Overall Survival by ERBB2 Status Within Race and Ethnicity Among Patients With TNBC in the NCDB (2010-2019) eFigure 4. Unadjusted Overall Survival by Race and Ethnicity Stratified for ERBB2 Status and Overall Response eTable 4. Adjusted Overall Survival Among All Patients With TNBC Stratified by Race and Ethnicity [file jamanetwopen-e2514864-s001.pdf]

Supplementary Online Content

Botty van den Bruele A, Crowell K-A, Thomas SM, et al. *ERBB2*-low expression by race and ethnicity among patients with triple-negative breast cancer. *JAMA Netw Open.* 2025;8(6):e2514864. doi:10.1001/jamanetworkopen.2025.14864

eFigure 1. Flow Chart of Cohort of Patients with TNBC

eMethods. Race and Ethnicity Reporting in NCDB

eTable 1. Logistic Regression Predicting Low vs Zero *ERBB2* Expression (25 843 Patients;13,130 Events) Among Patients With TNBC in the NCDB (2010-2019)

eTable 2. Rates of Pathologic Complete Response by *ERBB2* Status within Race and Ethnicity

eFigure 2. Unadjusted Overall Survival by *ERBB2* Status among Patients With TNBC in the NCDB (2010-2019)

eTable 3. Total Number of Patients for Each Race and Ethnicity by *ERBB2* Status and Number of Deaths and Censored Percent with Survival Rate

eFigure 3. Unadjusted Overall Survival by *ERBB2* Status within Race and Ethnicity Among Patients With TNBC in the NCDB (2010-2019)

eFigure 4. Unadjusted Overall Survival by Race and Ethnicity Stratified for *ERBB2* Status and Overall Response

eTable 4. Adjusted Overall Survival among All Patients With TNBC Stratified by Race and Ethnicity

This supplementary material has been provided by the authors to give readers additional information about their work.

© 2025 Botty van den Bruele A et al. *JAMA Network Open*.

eFigure 1. Flow Chart of Cohort of TNBC Patients

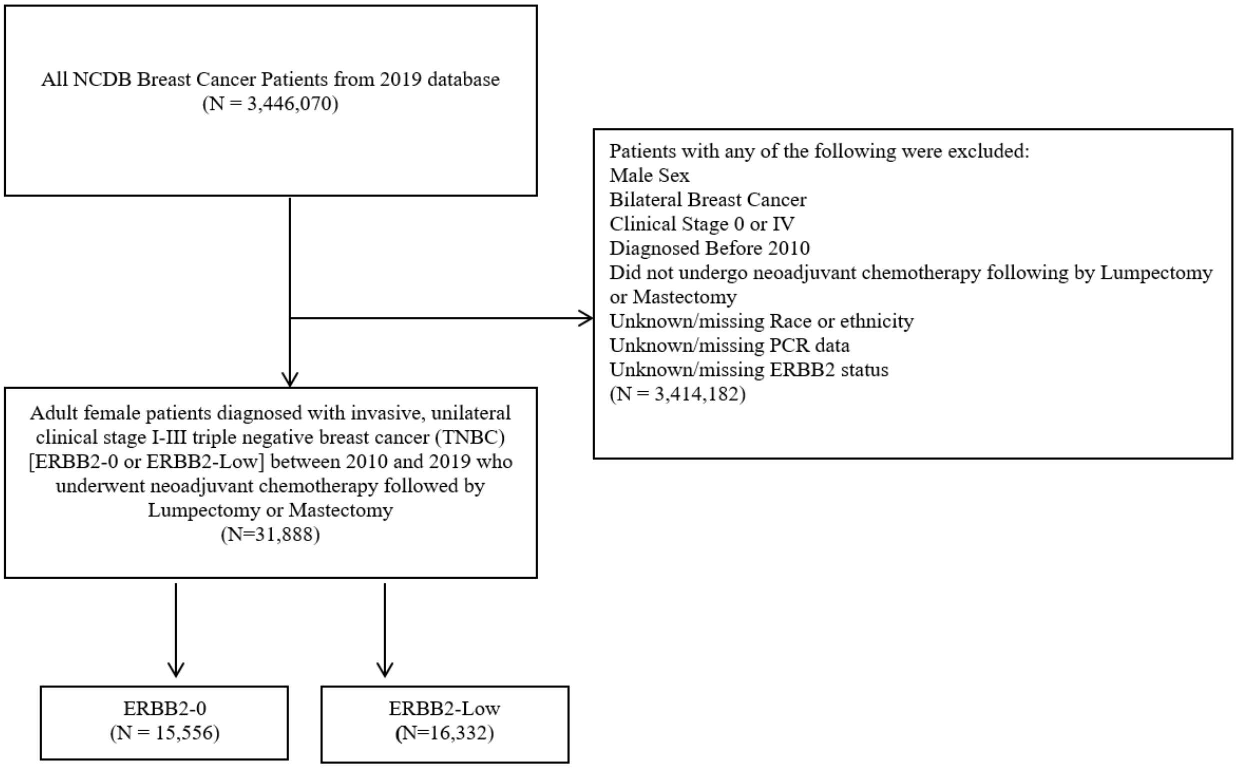

© 2025 Botty van den Bruele A et al. *JAMA Network Open*.

eMethods

Race and ethnicity are recorded in the NCDB based on the Standards for Oncology Registry Entry (STORE) guidelines. The STORE guidelines allow for up to 5 races to be entered for a given patient, however, only the primary race as entered into the first race field is included in the NCDB as the patient's race. According to the STORE guidelines:

- If the person is multiracial and one of the races is white, code the other race(s) first with white in the next race field.
- If the person is multiracial and one of the races is Hawaiian, code Hawaiian as Race 1, followed by the other race(s).

The NCDB includes a variable for race ("RACE") and a variable for ethnicity ("SPANISH_HISPANIC_ORIGIN"). The race variable includes 28 possible values in addition to "Other" and "Unknown" (see table below). Because of the large number of values, it is not feasible to list all race options within our manuscript. As is common practice, we have instead defined race as White (01 from the table below), Black (02), Asian (04, 05, 06, 08, 10, 11, 12, 13, 14, 15, 16, 17, 96), and Other (03, 07, 20, 21, 22, 25, 26, 27, 28, 30, 31, 32, 97, 98). As recommended by JAMA (https://jamanetwork.com/journals/jama/fullarticle/2783090), we have opted to report race and ethnicity as an aggregate unified term: Hispanic, Non-Hispanic Asian, Non-Hispanic Black, Non-Hispanic White, and Other.

Code	Definition
01	White
02	Black
03	American Indian, Aleutian, or Eskimo
04	Chinese
05	Japanese
06	Filipino
07	Hawaiian
08	Korean
10	Vietnamese
11	Laotian
12	Hmong
13	Kampuchean (including Khmer and Cambodian)
14	Thai
15	Asian Indian or Pakistani, NOS (formerly code 09)
16	Asian Indian
17	Pakistani
20	Micronesian, NOS
21	Chamorran
22	Guamanian, NOS
25	Polynesian, NOS
26	Tahitian
27	Samoan
28	Tongan
30	Melanesian, NOS
31	Fiji Islander
32	New Guinean
96	Other Asian, including Asian, NOS and Oriental, NOS
97	Pacific Islander, NOS
98	Other
99	Unknown

© 2025 Botty van den Bruele A et al. *JAMA Network Open*.

eTable 1. Logistic regression predicting ERBB2 Low vs ERBB2-0 (N = 25843, events = 13130) status among Patients with TNBC in the NCDB (2010 – 2019)

	Odds Ratio (95% CI)	*P*-Value	Overall *P*-Value
**Race and Ethnicity**			.006
Non-Hispanic White	REF		
Hispanic	0.84 (0.76 – 0.92)	.003	
Non-Hispanic Asian	0.98 (0.85 – 1.12)	.77	
Non-Hispanic Black	0.99 (0.93 – 1.01)	.78	
Other	1.11 (0.87 – 1.43)	.39	
**Age (years)**			.001
50+	REF		
<50	0.916 (0.87 – 0.97)	.001	
**Charlson/Deyo Comorbidity Score**			.86
0	REF		
1	1.01 (0.93 – 1.09)	.84	
≥2	0.96 (0.83 – 1.12)	.63	
**Income Level**			.18
<$48,000	REF		
≥48,000	0.96 (0.90 – 1.02)	.18	
**Insurance Type**			.02
Private	REF		
Government	1.08 (1.92 – 1.14)	.005	
None	1.04 (0.90 – 1.20)	.62	
**Education Level**			.03
High School Graduation Rate ≤87%	REF		
High School Graduation Rate >87%	1.07 (1.00 – 1.14)	.03	
**Facility Type**			<.001
Academic/Research	REF		
Community Cancer Program	1.16 (1.04 – 1.30)	.01	
Comprehensive Community Cancer Program	1.20 (1.14 – 1.27)	<.001	
Integrated Network Cancer Program	1.13 (1.06 – 1.21)	<.001	
**Facility Location**			<.001
South	REF		
Midwest	0.99 (0.93 – 1.05)	.75	
Northeast	0.88 (0.82 – 0.95)	<.001	
West	1.09 (1.01 – 1.18)	.04	
**Histology**			.001
Ductal	REF		
Lobular	0.93 (0.82 – 1.06)	.28	
Other	0.83 (0.75 – 0.92)	<.001	
**Clinical T-Category**			.16
cT1	REF		
cT0/is	0.84 (0.43 – 1.63)	.61	
cT2	1.07 (1.00 – 1.14)	.04	
cT3	1.09 (1.00 – 1.19)	.03	
cT4	1.10 (0.99 – 1.21)	.08	
**Clinical N-Category**			<.001
cN0	REF		
cN1	1.15 (1.09 – 1.21)	<.001	
cN2	1.03 (0.93 – 1.14)	.62	

© 2025 Botty van den Bruele A et al. *JAMA Network Open*.

cN3	1.19 (1.07 – 1.34)	0.002	
**Grade**			<.001
1	REF		
2	0.98 (0.74 – 1.32)	0.92	
3	0.86 (0.64 – 1.14)	0.28	

© 2025 Botty van den Bruele A et al. *JAMA Network Open*.

eTable 2. Rates of Pathologic Complete Response by ERBB2 status within Race and Ethnicity

ERBB2 Status and Response		Hispanic N=827 N (%)	Asian N =305 N (%)	Black N = 2038 N (%)	White N = 6196 N (%)	Other N = 96 N (%)
ERBB2-zero	pCR	462 (33.21)	170 (32.14)	1006 (27.06)	3101 (31.74)	48 (32.21)
ERBB2-low	pCR	365 (30.75)	135 (24.59)	1032 (26.30)	3095 (29.49)	48 (27.12)
P value		.01	.05	.56	.93	1.00

Abbreviations: pCR, pathologic complete response

© 2025 Botty van den Bruele A et al. *JAMA Network Open*.

eFigure 2. Unadjusted Overall Survival by ERBB2 Status among Patients with TNBC in the NCDB (2010-2019)

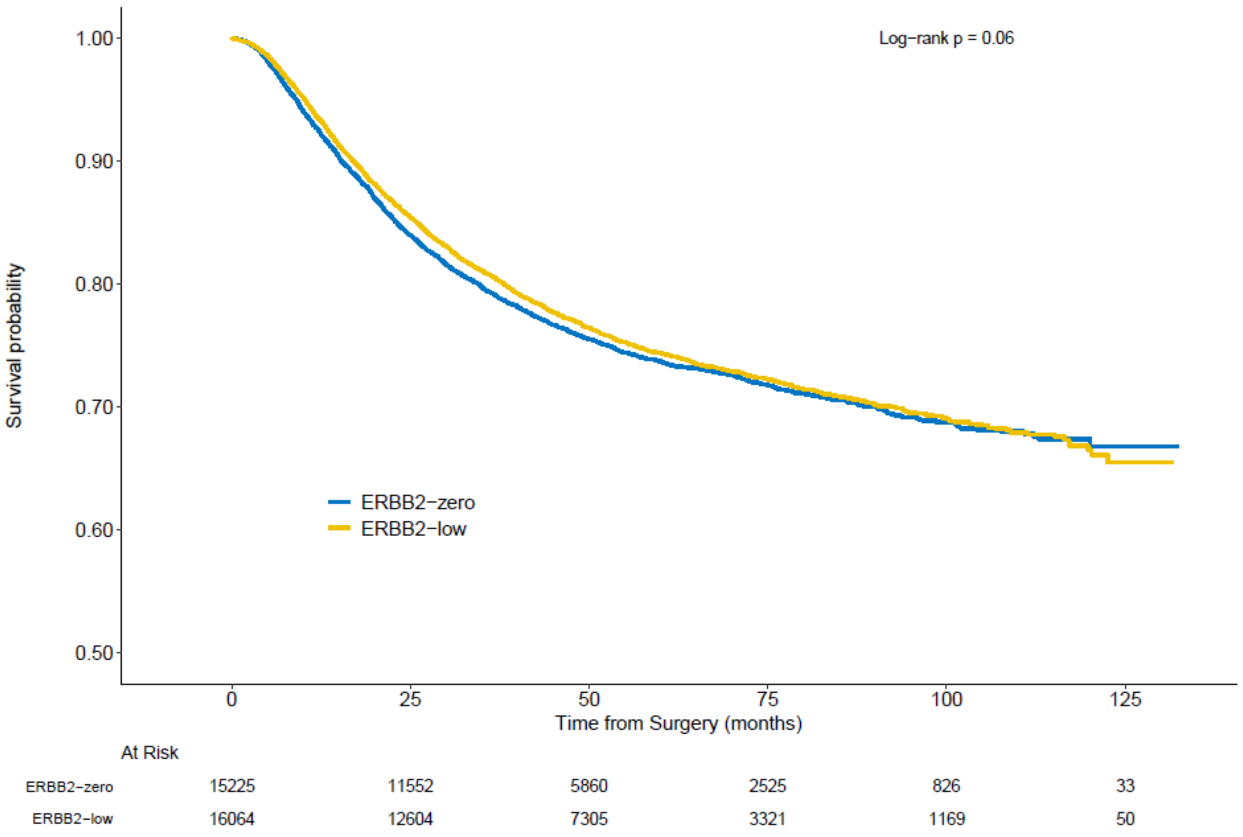

© 2025 Botty van den Bruele A et al. *JAMA Network Open*.

eTable3. Total Number of Patients for each Race and Ethnicity by ERBB2-status and Number of Deaths and Censored Percent with Survival Rate

Summary of the Number of Censored and Uncensored Values						Survival Rate (95% CI)			
						12 months	36 months	60 months	120 months
**ERBB2-Zero**	**Race**	**Total**	**Failed**	**Censored**	**Percent Censored**				
	**Hispanic**	1354	251	1103	81.46	0.934 (0.919-0.946)	0.829 (0.806-0.848)	0.781 (0.754-0.805)	0.756 (0.717-0.791)
	**Non-Hispanic Asian**	512	89	423	82.62	0.958 (0.936-0.972)	0.843 (0.805-0.873)	0.792 (0.746-0.83)	0.713 (0.615-0.789)
	**Non-Hispanic Black**	3643	971	2672	73.35	0.91 (0.9-0.919)	0.759 (0.744-0.773)	0.705 (0.688-0.721)	0.642 (0.615-0.667)
	**Non-Hispanic White**	9574	2259	7315	76.40	0.93 (0.924-0.935)	0.799 (0.791-0.808)	0.741 (0.731-0.751)	0.677 (0.66-0.694)
	**Other**	142	31	111	78.17	0.949 (0.896-0.975)	0.839 (0.764-0.892)	0.759 (0.667-0.829)	0.592 (0.362-0.763)
**Total**		15225	3601	11624	76.35				
**ERBB2-Low**	**Race**	**Total**	**Failed**	**Censored**	**Percent Censored**				
	**Hispanic**	1166	240	926	79.42	0.943 (0.928-0.955)	0.829 (0.805-0.85)	0.768 (0.739-0.794)	0.71 (0.659-0.755)
	**Non-Hispanic Asian**	535	75	460	85.98	0.96 (0.94-0.974)	0.89 (0.859-0.914)	0.837 (0.796-0.87)	0.801 (0.742-0.848)
	**Non-Hispanic Black**	3861	1038	2823	73.12	0.925 (0.916-0.933)	0.777 (0.763-0.791)	0.71 (0.694-0.725)	0.652 (0.628-0.674)
	**Non-Hispanic White**	10335	2438	7897	76.41	0.938 (0.933-0.943)	0.814 (0.806-0.821)	0.75 (0.74-0.759)	0.66 (0.637-0.681)
	**Other**	167	40	127	76.05	0.915 (0.861-0.949)	0.763 (0.686-0.823)	0.728 (0.644-0.796)	0.7 (0.599-0.781)
**Total**		16064	3831	12233	76.15				

© 2025 Botty van den Bruele A et al. *JAMA Network Open*.

eFigure 3. Unadjusted Overall Survival by ERBB2 Status within Race and Ethnicity among Patients with TNBC in the NCDB (2010-2019)

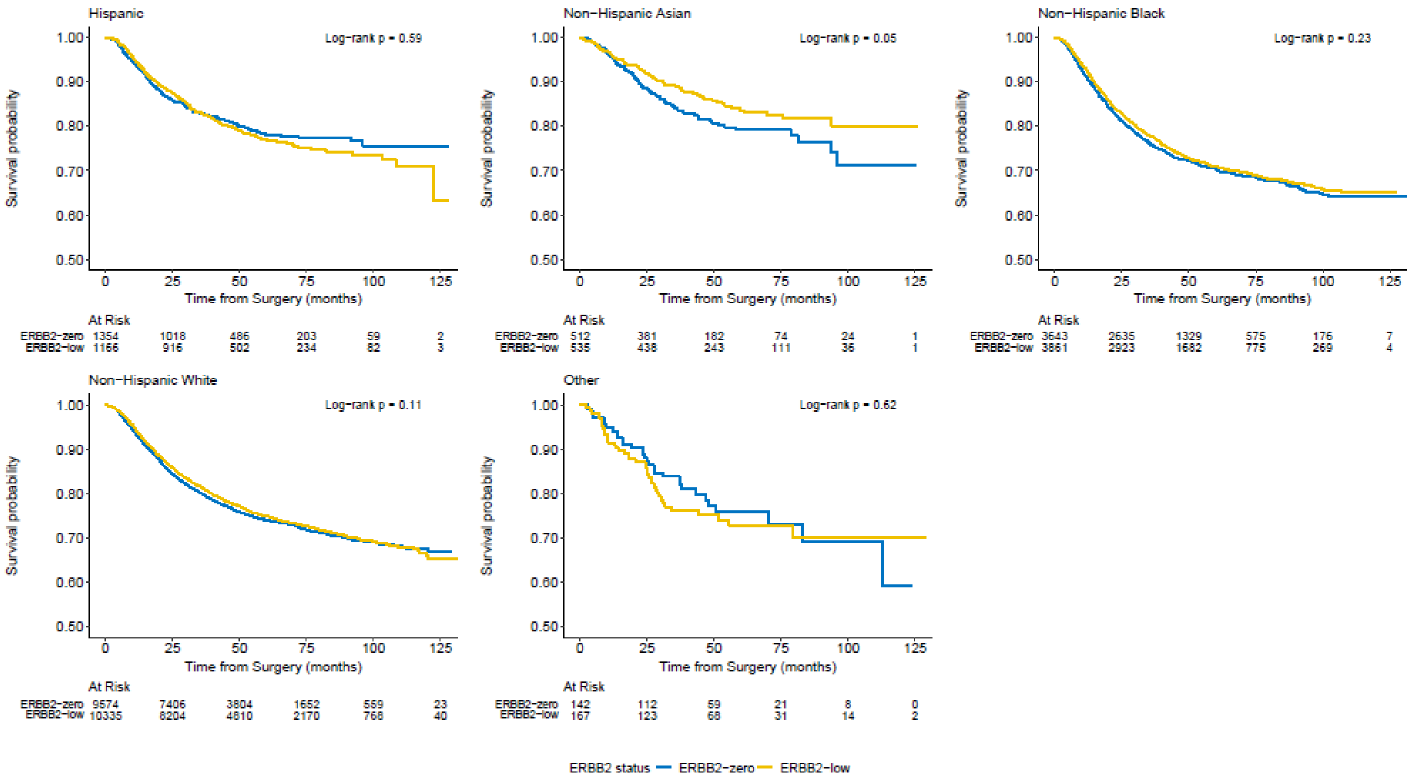

© 2025 Botty van den Bruele A et al. *JAMA Network Open*.

eFigure 4. Unadjusted Overall Survival by Race and Ethnicity Stratified for ERBB2 Status and Overall Response

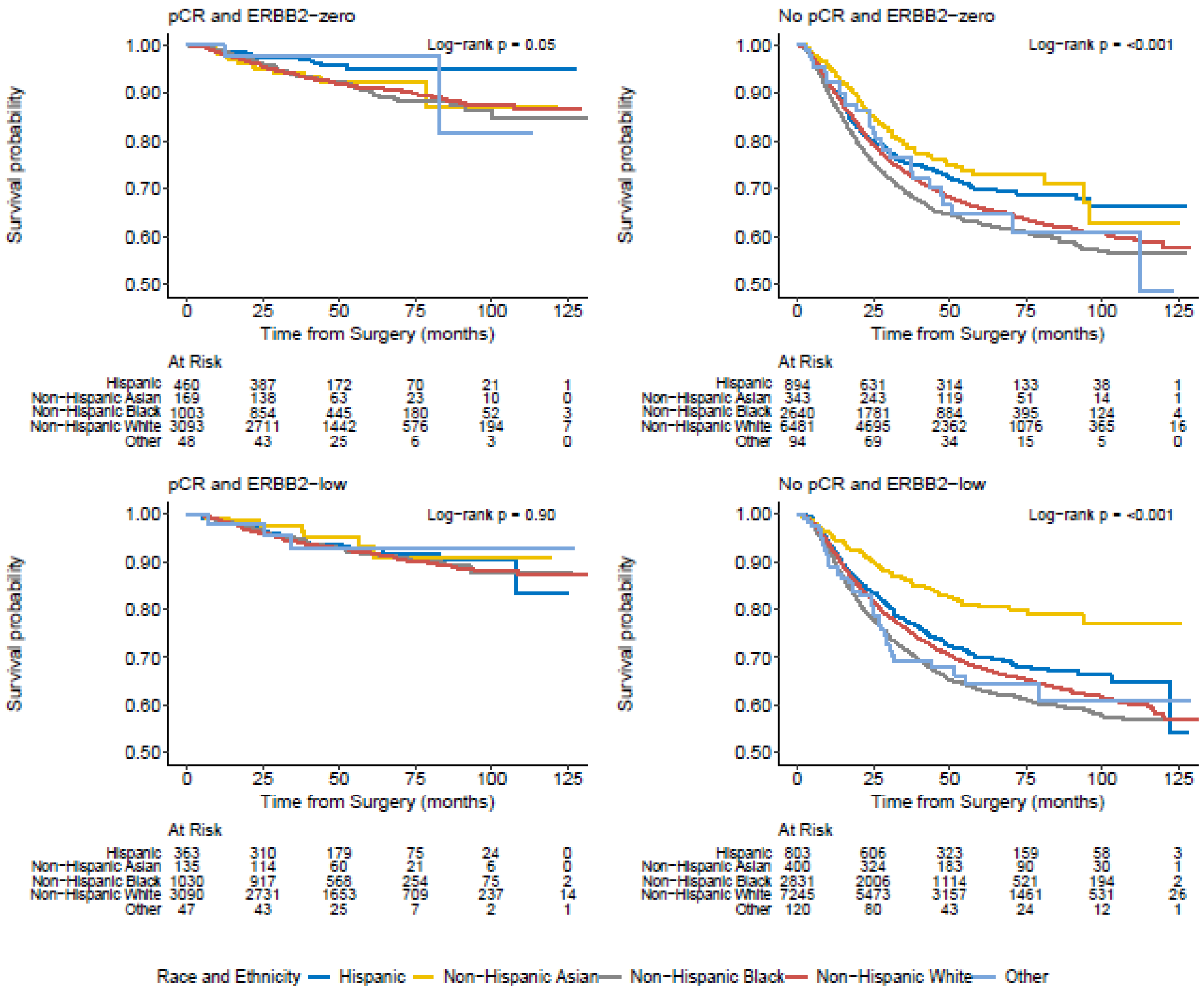

© 2025 Botty van den Bruele A et al. *JAMA Network Open*.

eTable 4. Adjusted Overall Survival among All Patients with TNBC Stratified By Race and Ethnicity

	Hispanic[1] (N = 1899, events = 341)			Non-Hispanic White[2] (N = 14402), events = 3326			Non-Hispanic Black[3] (N = 5446), events = 1455			Non-Hispanic Asian[4] (N = 1047), events =164)			Other (N = 293)[5], events =65		
	**Hazard Ratio (95% CI)**	***P*-Value**	**Overall *P*-Value**	**Hazard Ratio (95% CI)**	***P*-Value**	**Overall *P*-Value**	**Hazard Ratio (95% CI)**	***P*-Value**	**Overall *P*-Value**	**Hazard Ratio (95% CI)**	***P*-Value**	**Overall *P*-Value**	**Hazard Ratio (95% CI)**	***P*-Value**	**Overall *P*-Value**
**ERBB2 Status**			.86			<.001			.05			.02			.79
ERBB2 – zero	REF			REF			REF			REF			REF		
ERBB2 - low	1.02 (0.82 – 1.30)	.86		0.85 (0.79–0.92)	<.001		0.89 (0.79–1.00)	0.05		0.69 (0.50–0.95)	0.023		1.07 (0.66–1.73)	0.79	
**Overall Response**			<.001			<.001			<.001			<.001			<.001
No Response	REF														
Complete Response	0.17 (0.11 – 0.26)	<.001		0.34 (0.31–0.39)	<.001		0.25 (0.20–0.30)	<.001		0.36 (0.24–0.56)	<.001		0.12 (0.05–0.34)	<.001	
**Age (years)**			.10			<.001			.06			.008			.76
50+	REF			REF			REF			REF			REF		
<50	1.19 (0.96 – 1.48)	.10		0.85 (0.78–0.92)	<.001		0.89 (0.78–1.00)	0.06		0.64 (0.46-0.89)	0.008		0.93 (0.57–1.51)	0.76	
**Charlson/Deyo Comorbidity Score**			<.001			.27			<.001			.07			
0	REF			REF			REF			REF			REF		
1	1.35 (0.97 – 1.89)	.07		1.06 (0.93–1.20)	.41		1.32 (1.13–1.54)	<.001		0.95 (0.58–1.56)	.84				
≥2	3.30 (2.02 – 5.39)	<.001		1.19 (0.93–1.52)	0.160		1.41 (1.04–1.90)	0.027		3.03 (1.16–7.89)	0.02				
**Clinical T-Stage**			<.001			<.001			<.001			<.001			
cT1	REF			REF			REF			REF			REF		
cT0/is	0.00 (0.00 – 0.00)	<.001		1.93 (0.84–4.43)	.008		0.30 (0.07–1.18)	0.09		0.00 (0.00–0.00)	<.001				
cT2	1.03 (0.73 – 1.46)	.84		2.41 (2.16–2.69)	<.001		2.13 (1.78–2.54)	<.001		1.27 (0.76–2.11)	.36				

© 2025 Botty van den Bruele A et al. *JAMA Network Open*.

cT3	1.17 (0.81 – 1.70)	.40		5.87 (5.07–6.79)	<.001		5.29 (4.13–6.78)	<.001		2.21 (1.22–3.99)	.009				
cT4	1.54 (0.96 – 2.46)	.07		4.27 (3.64–5.01)	<.001		5.57 (4.29–7.24)	<.001		3.29 (1.76–6.16)	<.001				
**Clinical N-Stage**			<.001			<.001			<.001			<.001			
cN0	REF			REF			REF			REF			REF		
cN1	1.84 (1.37 – 2.48)	<.001		1.64 (1.50–1.79)	<.001		1.72 (1.50–1.98)	<.001		2.15 (1.46–3.17))	<.001				
cN2	2.13 (1.40 – 3.24)	<.001		1.92 (1.65–2.25)	<.001		2.23 (1.80–2.77)	<.001		1.34 (0.57–3.14)	.51				
cN3	2.54 (1.60 – 3.96)	<.001		2.70 (2.33–3.13)	<.001		2.30 (1.84–2.86)	<.001		3.28 (1.89–5.69)	<.001				
**Grade**			.14			.03			.24						.29
1	REF			REF			REF			REF			--	--	
2	2.84 (0.43 – 18.4)	.27		1.00 (0.68–1.49)	.98		2.00 (0.87–4.58)	.10					0.68 (0.34–1.39)	.29	
3	2.11 (0.32 – 13.82)	.43		1.15 (0.77–1.71)	.48		2.06 (0.89–4.74)	.09					REF		
Tumor Size (cm)[+]				2.34 (2.17–2.53)	<.001	<.001	1.51 (1.45–1.57)	<.001	<.001						
Tumor Size × log(time)				0.71 (0.69–0.73)	<.001	<.001	0.81 (0.79–0.83)	<.001	<.001						

Abbreviations: CI, confidence interval; pCR, pathologic complete response; RD, residual disease; T, tumor; is, in situ; N, nodal
[1]Also adjusted for insurance status, facility type/location, education status, income level, community type, surgery type, radiation, time to treatment
[2,3]Also adjusted for insurance status, facility type/location, education status, income level, community type, surgery type, radiation, time to treatment, included time varying tumor to meet PH assumption
[4,5] No further adjustments due to number of events
[5]Grade 3 as reference group due to no patients in grade 1
[+]Model included a time-dependent interaction between tumor size*log(time); HR for interaction: White (0.71, 95%CI 0.69 – 0.73, *P* <.001), Black (0.81, 95% CI 0.79-0.83, *P*<.001).

© 2025 Botty van den Bruele A et al. *JAMA Network Open*.
