# Supplementary material for: ERBB2-Low Expression by Race and Ethnicity Among Patients With Triple-Negative Breast Cancer
Source: JAMA Netw Open. 2025 Jun 11;8(6):e2514864. doi: 10.1001/jamanetworkopen.2025.14864 (PMC12159773; doi:10.1001/jamanetworkopen.2025.14864)
Supplement: Supplement 2. — Data Sharing Statement [file jamanetwopen-e2514864-s002.pdf]

Data Sharing Statement

Botty van den Bruele. ERBB2-Low Expression by Race and Ethnicity Among Patients With Triple-Negative Breast Cancer. *JAMA Netw Open*. Published June 11, 2025. doi:10.1001/jamanetworkopen.2025.14864

Data
Data available: No

Additional Information
Explanation for why data not available: The data collected for this study is maintained by the American College of Surgeons as part of the National Cancer Database and can only be obtained through a direct request by an investigator associated with a Coc-accredited cancer program.
